# Characterising the application of the “progressive overload” principle of exercise training within cardiac rehabilitation: A United Kingdom-based community programme

**DOI:** 10.1371/journal.pone.0237197

**Published:** 2020-08-13

**Authors:** Alaa Khushhal, Simon Nichols, Sean Carroll, Grant Abt, Lee Ingle

**Affiliations:** 1 Department of Sport, Health & Exercise Science, University of Hull, Kingston-upon-Hull, United Kingdom; 2 Centre for Sport and Exercise Science, Sheffield Hallam University, Collegiate Campus, Sheffield United Kingdom; Teesside University/Qatar Metabolic Institute, UNITED KINGDOM

## Abstract

**Background:**

Recent concerns have cast doubt over the effectiveness of cardiac rehabilitation [CR] programmes for improving cardiorespiratory fitness [CRF] in patients with a history of cardiac disease in the United Kingdom [UK]. We aimed to characterise the weekly progression of exercise training dose over an 8-week Phase III CR programme as we felt this may be partly responsible for the lack of improvement in CRF reported in previous studies.

**Design:**

Observational study.

**Methods:**

We evaluated a community-based Phase III CR programme in the UK. During each training session, patients wore an Apple Watch and the weekly progression of exercise training dose/load was quantified. The analysis was based on 332 individual training sessions. Exercise intensity [% heart rate reserve] during the cardiovascular [CV] exercise training component [%HRR-CV], CV training duration; estimated changes in cardiorespiratory fitness [change in estimated metabolic equivalents (METs)]; session rating of perceived exertion [sRPE], sRPE training load [sRPE-TL], and exercise training impulse [TRIMP] were evaluated.

**Results:**

Thirty cardiac patients [83% male; age [SD] 67.0 [10.0] years; body mass index [SD] 28.3 [4.6] kg∙m^-2^] were recruited to an 8-week programme [16 sessions in total]. Bayesian repeated-measures ANOVA indicated anecdotal evidence for the alternative hypothesis for changes in %HRR-CV (BF_10_ = 0.61), sRPE (BF_10_ = 1.1), and change in estimated METs (BF_10_ = 1.2) during CR. Conversely, Bayesian repeated-measures ANOVA showed extreme evidence for changes in CV training duration (BF_10_ = 2.438e+26), TRIMP (BF_10_ = 71436), and sRPE-TL (BF_10_ = 779570).

**Conclusion:**

The key exercise training principle of progressive overload was only partially applied. Increases observed in exercise dose were due to increases in the duration of CV training, rather than combined with increases in exercise intensity [%HRR-CV and sRPE]. Accordingly, allied health professionals must ensure that exercise intensity is more consistently progressed to optimise the exercise stimulus and improvements in CRF and patient outcomes.

## Introduction

In 2016, an updated Cochrane review, [1] synthesising 63 international studies, concluded that compared to no exercise control, exercise-based cardiac rehabilitation (CR) reduced the risk of subsequent cardiovascular mortality, but not total mortality. Studies emanating from the United Kingdom (UK) have cast doubts over the survival benefits of CR programmes. Initially, the RAMIT trial [2], a large multi-center randomised controlled trial based in representative hospitals in England and Wales, compared 1,813 patients referred to comprehensive CR, or discharged to 'usual care' [without specific referral to CR]. The study found that CR following myocardial infarction (MI) had no important effect on mortality, cardiac or psychological morbidity, risk factors, health-related quality of life, or physical activity levels. On the basis of the findings, RAMIT investigators concluded that “the value of cardiac rehabilitation as practised in the UK is open to question” (p.637). More recently, Powell and colleagues [3] conducted a systematic review and meta-analysis to determine the effectiveness of exercise-based CR in terms of all-cause mortality, cardiovascular mortality, and hospital admissions from the year 2000 onwards, which include only trials which use a modern approach to medical management. They included 22 studies including 4,834 patients (mean age 59.5 years, 78.4% male). Their analysis indicated “conclusively” that the current approach to exercise-based CR has no effect on all-cause mortality or cardiovascular mortality, when compared to no-exercise control (p.1). Earlier support for these findings was reported by Sandercock et al [4] who conducted a UK-based multi-centre study to quantify changes in cardiorespiratory fitness (CRF) changes before and after CR. The above authors concluded that low exercise training volumes and small increases in CRF (0.52 metabolic equivalents) may partially explain the reported inefficacy of UK CR to improve patient mortality and morbidity.

Supervised CR programmes in the UK focus on an interval approach to training where patients’ training intensity toggles between cardiovascular (CV) training, and active recovery (AR) exercise. UK guidance indicates that patients should train at a fixed exercise intensity between 40–70% heart rate reserve (%HRR). [5,6] The 40% HRR threshold is cited as the lowest effective exercise intensity for improving CRF in patients undertaking CR. [6] Training at or above the ventilatory anaerobic threshold (VAT) induces physiological adaptation leading to improved CRF and other cardiovascular risk factors. [7,8] We recently showed that the fixed percentage method (%HRR) was poorly correlated with an objective, threshold-based approach incorporating the accurate determination of VAT. [9] In 112 referred cardiac patients, we found that VAT thresholds were identified outside of the 40–70% predicted HRR exercise training zone in 55% of patients. In the majority, the VAT occurred at an exercise intensity <40% HRR. These findings indicated that a fixed percentage range approach may be inaccurate for prescribing exercise dose in a large proportion of patients undertaking CR. Recently, in a detailed analysis of weekly CR training in one UK CR centre, we used digital technology to monitor and record week-by-week progression of individual training load. [10] This study was performed because we postulated that inadequate exercise dose may be partly responsible for emerging evidence that CR programmes may not be optimised for improving CRF in patients with CHD. We found that average mean training intensity achieved over eight weeks was below the lower limit of the minimal exercise training intensity guidelines (<40% HRR) for a Phase III CR programme. [10] If these findings are generalisable to other UK CR centres, they may be in part responsible for previous reports highlighting significant variability in effectiveness of UK CR services and poor CRF improvements observed from several prior investigations.

The accronym ‘SPORT’ can be used to describe the five main principles of training, including specificity, progression, overload, reversibility, and tedium. We combined two of these principles into ‘progressive overload’ to examine the hypothesis that allied health professionals were not consistently applying the principle of progressive overload to cardiac patients undertaking a community-based Phase III CR programme. Insufficient progressive overload may be one of the reasons for the variability in CR outcomes, including a lack of improvement in CRF within UK programmes. Previous work in athletic performance settings has shown that the minimum increase in weekly training load should be ≥10% to gain significant adaptations and subsequent improvements in physical performance. [11] In cardiac populations, the proposed weekly increase in training load has yet to be clearly established. The aim of the study was to characterise the weekly progression of exercise dose over an 8-week Phase III CR programme.

## Materials and methods

### Ethical approval

The study was approved by the North West National Health Service (NHS) Research Ethics Committee (REC ref: 16/NW/0419) and institutional ethics committee prior to commencement of the study. All patients provided written informed consent before participating in the trial. Inclusion and exclusion criteria have been reported previously. [10] Patients were referred from their general practitioner, practice nurse or hospital doctor to a Phase III CR programme based in the North Eastern region of England.

### Cardiac rehabilitation programme

The 8-week Phase III CR programme contained 16 sessions in total [twice per week]. This was a physiotherapist-led service and the ratio of staff to patients was always 3 or 4: 1. Prior to each training session, a 10–15 minute warm-up was included, followed by a circuit programme based on an interval training approach. Nine exercise stations were performed of which five were cardiovascular (CV) and four were active recovery (AR) exercises. The specific exercise stations performed have been reported previously. [10] Patients were advised not to drink caffeine for at least 3 hours before the start of the exercise classes. Session RPE was ascertained immediately after the end of each training session. The CV stations were designed to be conducted within the patients’ individualised heart rate training zone between 40–70% of heart rate reserve, in line with national guidelines. [5,6] This was calculated using the following equation [12,13]:

MaximumHR=206–(agex0.7)

A symptom-limited submaximal exercise test was conducted on a cycle ergometer at baseline and following the 8-week CR programme. Changes in submaximal workload and estimated metabolic equivalents (METs) was recorded.

### Observational study protocol

A study investigator (AK) met and invited patients to participate in the study prior to their initiation in the Phase III, 8-week exercise class. At this point, it was clarified to patients that the only distinction between being enrolled in the study and undertaking the standard community-based CR programme was that they would be required to wear an Apple watch during their participation in the structured 8-week programme. During each exercise training session, patients wore an Apple Watch (Series 0 or 2, Watch OS2.0.1, Apple Inc., California, USA) on their left wrist, with the exception of one patient, who wore it on the right wrist due to an existing tattoo on the left. Each Apple Watch was connected via Bluetooth to an iPhone 5s or iPhone 6 (Apple Inc., California, USA). We used the ‘Workout’ app, which is the standard exercise app on the Apple watch to measure heart rate every 5 seconds. A bespoke iPhone app was written by one of the co-authors [GA] to extract the raw HR and sampling time data from the ‘Health’ database on the paired iPhone. The bespoke app was written using the Swift 2.1 language in Xcode 7.2.1, utilising the methods supplied by the HealthKit framework (Apple Inc., California, USA). The Apple watch uses photoplethysmography (PPT) to measure HR. PPT is a non-invasive measure and uses a sensor to note changes in blood flow to measure heart rate. [14] We recently revealed that the Apple watch heart rate sensor has good validity during jogging and running, and very good validity during walking activities. It is clear, however, that the validity of the Apple watch for measuring heart rate reduces as exercise intensities increases and particularly during exercise with rapid arm movements. [15] Once a patient was paired with a specific Apple watch they would continue to use the same watch for the entirety of the study.

### Measurements of exercise dose/load to determine weekly progression

Whilst we recorded all phases of the exercise training including the warm up and cool-down phases, both of these phases were not included in the final analysis. We were only concerned with the %HRR achieved during the CV and AR stations for each training session for each patient. Each CV and AR station consisted on one minute of exercise training. In order to calculate weekly changes in individual exercise training dose/load, we measured the following variables:

1. Exercise intensity: based on weekly changes in HRR during CV training (%HRR-CV).
2. Exercise duration: based on weekly changes in the duration of the CV component of exercise training (minutes and percentage change).
3. Exercise dose: based on weekly changes in the TRIMP measure of exercise training dose. [16] Banister’s training impulse (TRIMP) is a method of measuring internal load by combining exercise intensity based on changes in mean heart rate with exercise duration. [16] Banister’s TRIMP was calculated for each patient’s weekly training sessions using the following formula:

Exercise duration (mins) * mean heart rate * y; where y is a weighting factor.

4 We measured session rating of perceived exertion (sRPE), where each patient rated his/her exercise intensity subjectively at the end of every training session recalling their RPE for the entire session (Foster scale 0 to 10); where 0 is rest and 10 is maximal. [17]
5 In addition, we also calculated the weekly changes in session RPE training load (sRPE-TL) which is the product of session rating of perceived exertion (sRPE) multiplied by training session duration.
6 From the symptom-limited, submaximal cycle ergometer test we measured change in CRF, as estimated METs (baseline to 8-weeks later).

### Statistical analysis

Data analysis was conducted in JASP 0.10.2 [18] and SPSS Version 26 [19] using Bayesian statistical methods. We only included patient data in our final analysis if programme adherence was ≥75% (completed a minimum of 12 of 16 training sessions). Data residuals for each model described below were visually examined using Q-Q plots and found to be approximately normal. JASP does not provide the option to examine the assumption of sphericity when doing a Bayesian repeated-measures ANOVA, so the assumption was examined and dealt with in two steps. First, we conducted a frequentist repeated-measures ANOVA and associated sphericity tests in JASP to obtain estimates of epsilon. Second, if Mauchly’s test of sphericity was significant and the mean epsilon across Greenhouse-Geisser and Huynh-Feldt was substantially below 1, we proceeded to conduct a Bayesian repeated-measures ANOVA in SPSS. SPSS provides an adjusted Bayes factor [BF] when Mauchly’s test is significant. For %HRR-CV, TRIMP, and sRPE-TL, the assumption of sphericity was violated, so for those variables we report the adjusted BF_10_ values from SPSS. For all other variables the data did not violate the assumption of sphericity so the BF_10_ values from JASP are reported. Below we describe the statistical analysis for the following comparisons across each of the eight weeks of training:

1. Comparing mean %HRR-CV.
2. Comparing mean CV training duration.
3. Comparing mean TRIMP.
4. Comparing mean sRPE.
5. Comparing mean sRPE-TL.
6. Further, comparing pre and post estimated changes in CRF [estimated METs].

We examined the changes in mean %HRR-CV, mean sRPE, mean CV training duration, mean TRIMP, and mean sRPE-TL across each of the eight weeks of training both in the form of a hypothesis test using BFs and as a parameter estimation for the posterior distribution of the standardised difference. For model comparison we used BFs which quantify the relative predictive performance of the null hypothesis (H_0_) compared to the alternative hypothesis (H_1_). For each analysis we conducted a Bayesian repeated-measures ANOVA, which is equivalent to a Bayesian linear mixed-effects model where the individual intercepts were allowed to vary for participants, using a two-sided alternative hypothesis because it is unknown if there was progressive overload in these measures across the training programme. Where BF_10_ suggested strong evidence for H_1_, post-hoc pairwise comparisons of the standardised differences [median Cohen’s *d*–that is, the median value of the posterior distribution] are reported along with the associated 95% credible interval for each paired difference. Given our uncertainty in the effects, we assigned a flat prior with scale r = 0.5 for fixed effects and 1 for random effects. Subject was considered as a random effect. The repeated-measures ANOVAs conducted in SPSS used Rouder’s mixed design to estimate BFs, which uses multivariate generalizations of the Cauchy distribution as the prior for standardized effect size, and a non-informative prior for variance. To examine the change (if any) in CRF (estimated METs) we used a two-sided Bayesian paired-sample t-test with a broad weakly informative prior using a zero-centred Cauchy distribution with scale r = 1/√2. Although the BF is a continuous measure of evidence for H_1_ relative to H_0_ with higher values indicative of increasingly stronger support for H_1_, the classification scheme of Lee and Wagenmakers [20] is useful for interpretation. In this classification scheme a BF_10_ between one and three is indicative of anecdotal evidence, between three and 10 is indicative of moderate evidence, between 10 and 30 is indicative of strong evidence, between 30 and 100 is indicative of very strong evidence, and above 100 is indicative of extreme evidence. To describe the magnitude of the standardised effect size we use the classification scheme of Cohen, [21] with 0.2, 0.5 and 0.8 representing small, moderate, and large effects, respectively.

## Results

Thirty cardiac patients (83% male; age [SD] 67.0 [10.0] years; body mass index [SD] 28.3 [4.6] kg∙m^-2^) were recruited to the Phase III CR programme. Data was collected over an 18-month period between November 2017 and May 2019. Patients were prescribed beta-blockers (87%), statins (53%), ACE-inhibitors (40%), and aspirin (68%). Medications remained unchanged throughout the training intervention. Eleven patients included in the intervention had received a coronary artery bypass graft, seven had received percutaneous coronary intervention, and two patients had undergone a mitral valve replacement. We also recruited four patients following myocardial infarction, three patients diagnosed with chronic heart failure, and three patients diagnosed with coronary heart disease. Twenty one patients completed all 16 sessions of the Phase III CR programme; the remaining nine patients dropped out at various stages due to a deterioration in their health status, or were unable to attend due to personal or family reasons. Our analysis is based on 21 patients who had achieved our *a priori* programme adherence rate ≥75%. In total, following patient attrition and technical issues which affected data integrity, our final analysis is based on 332 individual exercise training sessions.

### Changes in %HRR-CV

**Fig 1:**
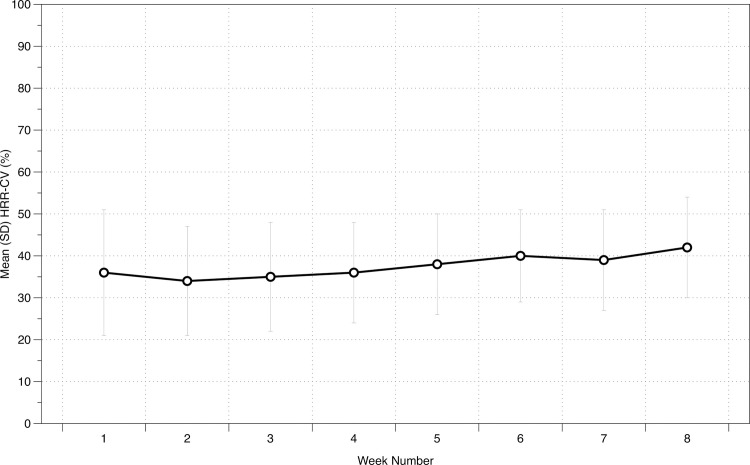
Weekly changes in exercise intensity based on %HRR-CV in cardiac patients undergoing 8-weeks of CR.

The mean (SD) % HRR during cardiovascular training for each of the eight training weeks is displayed in Table 1. The Bayesian repeated-measures ANOVA resulted in a BF_10_ of 0.61, indicating anecdotal evidence for the null hypothesis. Percentage weekly changes are displayed in Fig 1.

**Table 1:** Mean (SD) training intensity and training load collected over eight weeks of a community-based cardiac rehabilitation programme.

Measure	Week 1	Week 2	Week 3	Week 4	Week 5	Week 6	Week 7	Week 8
HRR-CV [%]	36 [14]	33 [13]	34 [13]	36 [12]	38 [12]	40 [11]	39 [12]	42 [12]
sRPE [AU]	2.6 [0.5]	2.9 [0.6]	3.0 [0.6]	3.0 [0.5]	2.9 [0.7]	3.1 [0.7]	3.0 [0.8]	3.3 [0.6]
CV time [min]	17 [6]	21 [8]	24 [9]	25 [9]	27 [8]	30 [7]	30 [8]	32 [8]
TRIMP [AU]	23 [17]	20 [13]	23 [17]	25 [18]	26 [20]	29 [18]	31 [20]	35 [23]
sRPE-TL [AU]	130 [43]	147 [39]	162 [51]	171 [42]	172 [57]	184 [58]	186 [68]	205 [52]

%HRR-CV = weekly mean percentage heart rate reserve during cardiovascular training; sRPE = session rating of perceived exertion [CR10]; CV time = weekly mean time spent doing cardiovascular training during any given training session; TRIMP = training impulse; sRPE-TL = session rating of perceived exertion training load [sRPE x training session duration]; AU = arbitrary units.

### Changes in CV training duration

**Fig 2:**
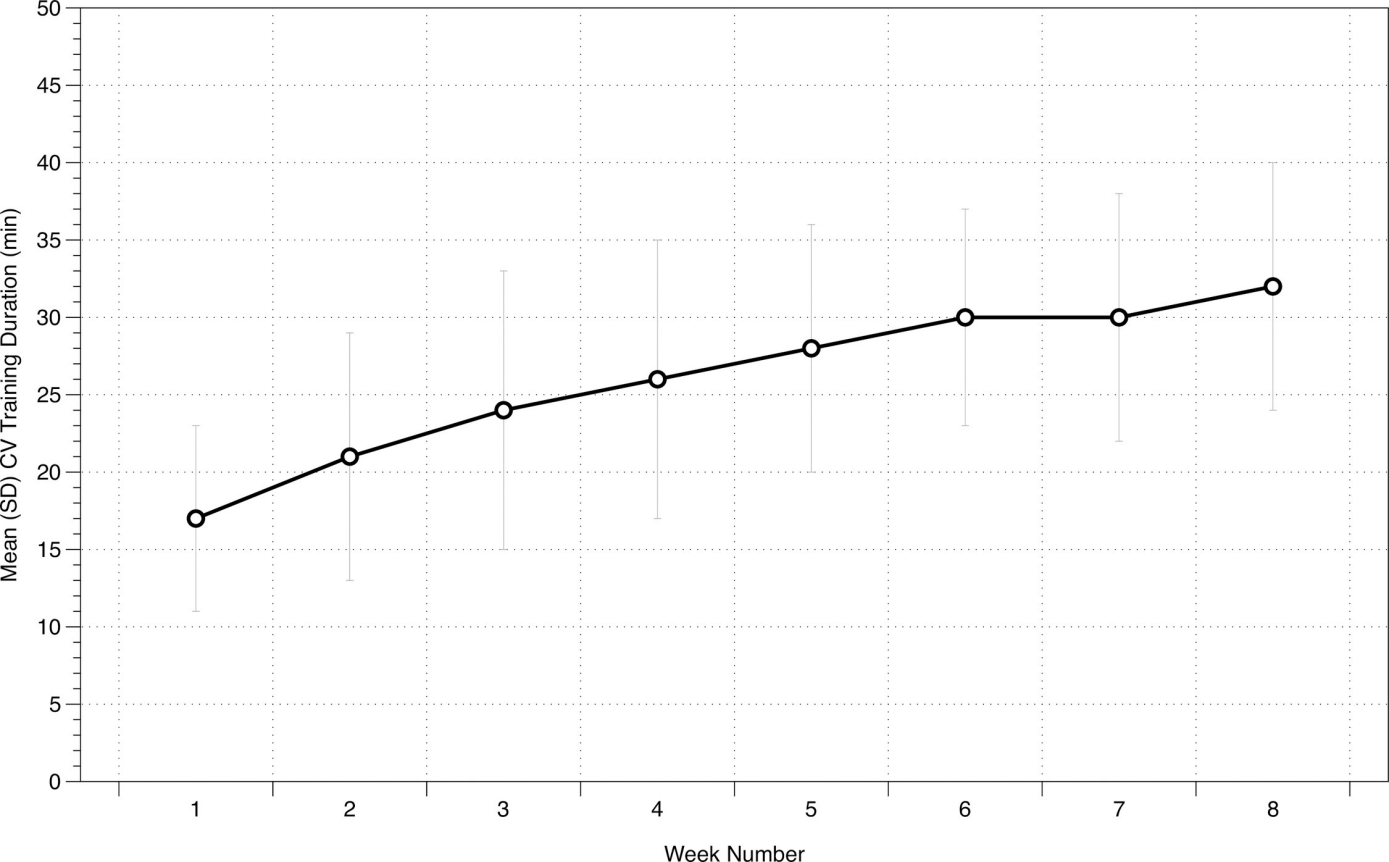
Weekly changes in exercise duration (minutes) in cardiac patients undergoing 8-weeks of CR. Our findings indicate extremely strong evidence for the alternative hypothesis of an effect of time on CV training duration.

The mean (SD) CV training duration for each of the eight training weeks is displayed in Table 1. The Bayesian repeated-measures ANOVA resulted in a BF_10_ of 2.438e+26, indicating extreme evidence for the alternative hypothesis of an effect of time on CV training duration (Fig 2).

### Changes in session RPE [sRPE]

The mean (SD) sRPE for each of the eight training weeks is displayed in Table 1. The Bayesian repeated-measures ANOVA resulted in a BF_10_ of 1.1, indicating anecdotal evidence for the alternative hypothesis of an effect of time on sRPE.

### Changes in Training Impulse [TRIMP]

**Fig 3:**
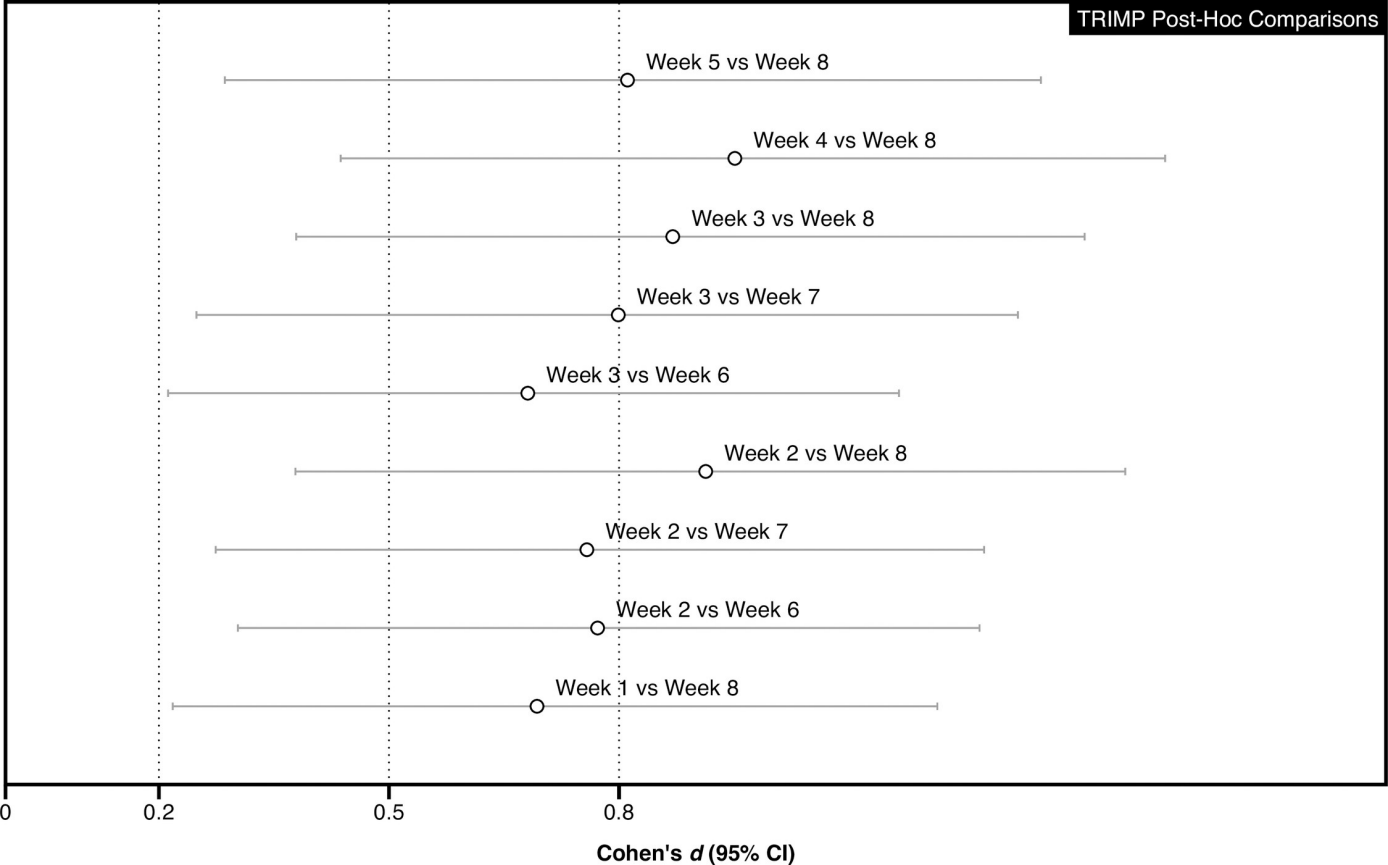
Post-hoc pairwise comparisons of the standardised differences (95% CI) for TRIMP.

The mean (SD) TRIMP for each of the eight training weeks is displayed in Table 1. The Bayesian repeated-measures ANOVA resulted in a BF_10_ of 71436, indicating extreme evidence for the alternative hypothesis of an effect of time on TRIMP. Post-hoc pairwise comparisons of the standardised differences (95% CI) between weeks are displayed in Fig 3.

### Changes in session RPE—Training Load [sRPE-TL]

**Fig 4:**
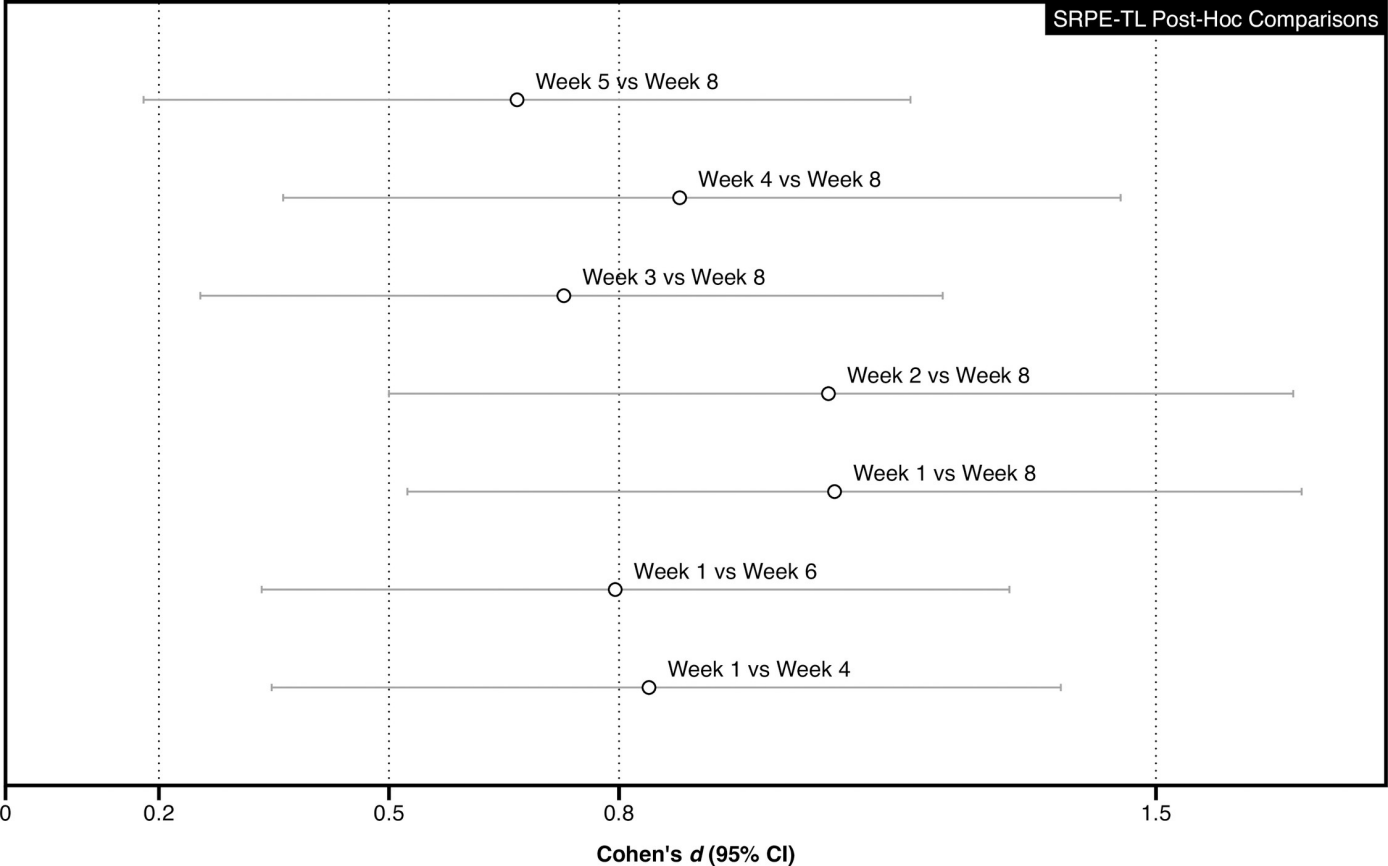
Post-hoc pairwise comparisons of the standardised differences (95% CI) for sRPE-TL.

The mean (SD) sRPE-TL for each of the eight training weeks is displayed in Table 1. The Bayesian repeated-measures ANOVA resulted in a BF_10_ of 779570, indicating extreme evidence for the alternative hypothesis of an effect of time on sRPE-TL. Post-hoc pairwise comparisons of the standardised differences (95% CI) between weeks are displayed in Fig 4.

### Estimated changes in cardiorespiratory fitness [Estimated METs]

The analysis was based on 16 patients as the remainder were lost to follow up. The mean (SD) estimated METs derived from pre and post cardiorespiratory fitness tests were 4.3 (1.9) and 5.2 (2.2) METS, respectively. The Bayesian paired samples t-test resulted in a BF_10_ of 1.2, indicating anecdotal evidence for the alternative hypothesis of an effect of time on estimated METs.

## Discussion

To our knowledge, this study is the first to characterise the weekly progression of exercise training dose/load over an 8-week Phase III community CR programme based in the UK. It appears that the key exercise training principle of progressive overload was partly applied, as demonstrated by the weekly increases in the duration of CV activity [and its surrogate measures] performed by cardiac patients undergoing community-based CR in the UK. However, allied health professionals must ensure that weekly increases in duration of the cardiovascular training component are supplemented by progression in exercise intensity, in order to optimise exercise training prescription and improve patient outcomes.

The duration of weekly CV training increased over the 8-week intervention, alongside sRPE-TL and our best measure of progressive overload, TRIMP, which also increased over the intervention [as CV training duration is a factor within its determination]. We found that CV training intensity was not “titrated” consistently over this period. Our study shows that there was anecdotal evidence for the alternative hypothesis of an effect of time for %HRR-CV over the 8-week intervention, indicating that training intensity was not individually titrated in accordance with established principles of exercise training (Fig 1). Previous work [11] in a sports performance setting highlighted that a minimum increase in weekly training load should be ≥10% in order to gain subsequent improvements in physical performance; whilst it is not clear what a minimum increase in training load should be in a clinical population, we did not observe an increase in CV training intensity (only CV training duration).

Although speculative, it is possible that the lack of meaningful increases in CV training intensity may be the consequence of conservative exercise prescription with the CR programme, and/or a lack of staff training and education. Guidance on exercise training progression is provided by the BACPR [5] and ACPICR [6] in their guidance and training documents/training modules. Emphasis of training should focus equally on increasing CV intensity and duration. We are hopeful that future interventions, such as a higher level qualification in the UK–the BACPR Certified Exercise Specialist, will focus on important training principles, such as progressive overload and its implementation, leading to improved service quality and exercise-related outcomes in the future. Clearly, wearable technology will continue to play an increasingly important role in supporting allied health professionals when monitoring exercise intensity and up-titrating individualised exercise dose/load during CR.

We did not measure external load (e.g. power output from the symptom-limited cycle ergometer test), so sub-optimal changes in internal intensity (%HRR) does not mean that patients were unable to increase their external load [e.g. power output] for the same internal intensity over time. We have previously argued that part of the problem associated with the perceived lack of effectiveness of some UK CR is programmes may be due to the widespread use of submaximal exercise testing e.g. 6-min walk test, incremental shuttle walk test, or the Chester step test [22] to determine programme effectiveness and to prescribe individual training thresholds. In the current CR programme, CRF was evaluated using a submaximal cycle ergometer test with the outcome measures used including changes in estimated METs. We recently showed that changes in estimated VO_2peak_ derived from the ACSM leg cycling equation is not an accurate surrogate for directly determined changes in VO_2peak_. We showed poor agreement between estimates of VO_2peak_ and directly determined VO_2peak_. Applying estimates of VO_2peak_ to determine CRF change may over-estimate the efficacy of CR and lead to a different interpretation of study findings. [23] There are very few centres in the UK which have adopted maximal cardiopulmonary exercise testing as routine for evaluating clinical populations and prescribing individualised exercise training loads. The outcomes of the CARE CR trial, which used criterion exercise testing methods to evaluate the effectiveness of a UK CR programme recently showed that in 70 patients with coronary heart disease, there was no changes in directly determined $\dot{V}O_{2\mathrm{peak}}$ at any time point following an 8-week CR programme. [24]

Our findings indicate that there was anecdotal evidence for a change in CRF (estimated METs) determined from the symptom-limited cycle ergometer test. We can conclude therefore that either [1] TRIMP and sRPE are not valid measures of training load for cardiac patients undertaking CR; [2] or the relative exercise intensity undertaken is insufficient for metabolic adaptations to improve cardiorespiratory fitness; or [3] the absolute increases in training load were not large enough to induce training adaptations, or [4] estimated CRF is not a sensitive enough measurement for changes in cardiorespiratory fitness to be identified. Whilst we are unaware of previous studies who have validated such measures (TRIMP and sRPE) in patients with coronary heart disease undertaking CR, Volterrani & Iellamo [25] have undertaken some initial validation work on TRIMP in patients with chronic heart failure. The same research group [26] have also argued that sRPE is a useful tool for long-term exercise prescription in cardiac patients. They estimated that to reach an effective weekly training load [e.g. around 400 AU], an effective exercise intervention should comprise 4 training sessions per week, with a session duration of 40–50 min at RPE 3–5 (Borg CR-10 scale). Compared to current practice, this estimated training load is significantly greater to what is currently available to cardiac patients undertaking CR in the UK. Our current study builds on our previous work [10] which showed that mean training intensity (average over 8 weeks) fell below the national recommended training intensity of 40% HRR (mean %HRR achieved = 37%) in 30 patients with CHD undertaking an 8-week Phase III CR programme in a single centre in the UK.

A limitation of our study is that our detailed evaluation of training intensity from a Phase III community-based CR programme is based on findings from a single NHS centre and a relatively small sample size and may not be representative of exercise training intensities or prescription methods undertaken in other UK centres. A previous report [27] has highlighted the high levels of inconsistent and variable quality of CR service provision in the UK. We utilised two models of Apple Watch in our study. It is possible that this technical issue may have introduced some degree of systematic error to our findings as we are not aware of the differences in technical specification between the models. We acknowledge that CR is a multi-dimensional intervention designed to address secondary prevention issues in this population. Our study focused purely on the exercise component, it was not designed to determine the effectiveness of CR for improving key outcome measures, for example, cardiorespiratory fitness. We have addressed this issue recently with the CARE CR study, highlighted previously. [24]

In conclusion, the exercise training principle of “progressive overload” was inconsistently applied- with weekly increases in the duration of CV activity (and its surrogate measures), but not its intensity component, performed by cardiac patients undergoing community-based CR in the UK. Allied health professionals must ensure that weekly increases in exercise intensity are consistently applied in order to optimise exercise training prescription and improve patient outcomes. These findings may be in part responsible for previous reports highlighting the lack of effectiveness of UK CR programmes.

## Supporting information

S1 Data Data collected for this investigation. (CSV)
